# Synthesis and Characterization of Chitosan-Containing ZnS/ZrO_2_/Graphene Oxide Nanocomposites and Their Application in Wound Dressing

**DOI:** 10.3390/polym14235195

**Published:** 2022-11-29

**Authors:** Yousef A. A. Alghuwainem, Mohamed Gouda, Mai M. Khalaf, Abraham Elmushyakhi, Manal F. Abou Taleb, Hany M. Abd El-Lateef

**Affiliations:** 1 Department of Veterinary Public Health and Care, College of Veterinary Medicine, King Faisal University, Al-Ahsa 31982, Saudi Arabia; 2 Department of Chemistry, College of Science, King Faisal University, Al-Ahsa 31982, Saudi Arabia; 3 Chemistry Department, Faculty of Science, Sohag University, Sohag 82524, Egypt; 4 Department of Mechanical Engineering, College of Engineering, Northern Border University, Arar 73213, Saudi Arabia; 5 Department of Chemistry, College of Science and Humanities, Prince Sattam Bin Abdulaziz University, Al-Kharj 11942, Saudi Arabia; 6 Department of Polymer Chemistry, National Center for Radiation Research and Technology (NCRRT), Egyptian Atomic Energy Authority, Nasr City, P.O. Box 7551, Cairo 11762, Egypt

**Keywords:** ZnS, ZrO_2_, graphene oxide, surface morphology, wound healing, cast method

## Abstract

The development of scaffold-based nanofilms for the acceleration of wound healing and for maintaining the high level of the healthcare system is still a challenge. The use of naturally sourced polymers as binders to deliver nanoparticles to sites of injury has been highly suggested. To this end, chitosan (CS) was embedded with different nanoparticles and examined for its potential usage in wound dressing. In detail, chitosan (CS)-containing zinc sulfide (ZnS)/zirconium dioxide (ZrO_2_)/graphene oxide (GO) nanocomposite films were successfully fabricated with the aim of achieving promising biological behavior in the wound healing process. Morphological examination by SEM showed the formation of porous films with a good scattering of ZnS and ZrO_2_ nanograins, especially amongst ZnS/ZrO_2_/GO@CS film. In addition, ZnS/ZrO_2_/GO@CS displayed the lowest contact angle of 67.1 ± 0.9°. Optically, the absorption edge records 2.35 eV for pure chitosan, while it declines to 1.8:1.9 scope with the addition of ZnS, ZrO_2_, and GO. Normal lung cell (WI-38) proliferation inspection demonstrated that the usage of 2.4 µg/mL ZnS/ZrO_2_/GO@CS led to a cell viability % of 142.79%, while the usage of 5000 µg/ mL led to a viability of 113.82%. However, the fibroblast malignant cell line exposed to 2.4 µg/mL ZnS/ZrO_2_/GO@CS showed a viability % of 92.81%, while this percentage showed a steep decline with the usage of 5000 µg/ mL and 2500 µg/mL, reaching 23.28% and 27.81%, respectively. Further biological assessment should be executed with a three-dimensional film scaffold by choosing surrounding media characteristics (normal/malignant) that enhance the selectivity potential. The fabricated scaffolds show promising selective performance, biologically.

## 1. Introduction

In spite of the recent significant development in both theoretical and experimental medical applications, the adjustment of biomaterials for the acceleration of wound healing is still a challenge [1,2]. This is explained by the strict necessities that must be fulfilled by the introduced materials [3,4]. These specifications include thermal, mechanical, optical, biological, and chemical properties. On one hand, the wound scaffolds should be adaptable and be able to cover of the curvatures of the body [5,6]. This could be achieved with polymeric materials [7,8]. In addition, the recommended materials have to be tailored with non-toxic raw materials with control of surface morphology in mind [9,10,11]. Obviously, the rough scaffolds offer high adhesion potential. In contrast, the desirable chemical behavior involves the ionic release property which may be reflected by high chemical stability [12]. Chitosan is a cationic polysaccharide with selective permeability behavior against toxic compositions such as CO as well as CO_2_ compounds [13]. It displays antibacterial activity and is nontoxic, biocompatible, biodegradable, and has antioxidant potential and film formation ability [14,15]. With respect to complications in CS-based bio-scaffolds, there has been a great demand for the perfection of new eco-friendly scaffolds [16]. Concerning the previous surveys, the dopant type and amount direct the original features that support the proposed application [17,18]. In this regard, nanoparticle-filled polymers offer a unique behavior owing to the special properties of nano-metric scale fillers [19,20,21,22].

Graphene oxide is a 2D arrangement of carbon atoms [19,20,21,22]. GO nano-filler enhances both mechanical and biocompatible performance [23,24,25]. Moreover, its oxyanion-releasing ability supports hydrophilicity and its biological applicability [26,27]. Selvi et al., 2016 have described the use of nanocomposites (NCs) as an excellent tactic for boosting physical, chemical, and biological behaviors. Regarding ZrO_2_-based NCs, recent studies have reported ZrO_2_/HAP as a highly bioactive ceramic material with significant cell adhesion potential, in addition to displaying excellent mechanical behaviors [28]. Additionally, the incorporation of ZrO_2_ into HAP NCs leads to the inhibition in corrosion reactions and promotes chemical stability [28]. With respect to the insertion of ZrO_2_ into the polymeric material, it has been reported that the incorporation of ternary phases (HAP/ZrO_2_/GO) into poly-lactic acid increases cell viability up to 98.2 ± 5%, and achieves an antibacterial potency of 69.2 ± 3.6 and 78.1 ± 4.5% against *E. coli* species and *S. aureus*, respectively [29]. Consequently, ZrO_2_-based NCs enhance the proliferation potential of human fibroblasts owing to the effect of its merging on the different behaviors of the resultant NCs that are tightly linked to the acceleration of the wound healing process.

On the other hand, zinc is crucially important for mankind and acts as a co-factor for transcription factors and enzymes which are specialized for growth, metabolism, and wound healing [30]. Zinc metalloproteins are vital in skin physiological cellular processes, including wound healing, promoting the re-epithelialization of skin cuts and fostering the proliferation of endothelial progenitor cells to promote angiogenesis [31]. Without zinc, the wound repair process might be delayed, and patients would suffer from unsealing wounds [30]. The element sulfur has been revealed to boost wound healing by demonstrating anti-inflammatory potential [32]. These two elements can be combined to create zinc sulfide (ZnS), which offers a wide band-gap energy of 3.7 eV and has been widely applied in biomedicine [30]. Moreover, different characterization techniques are important in biomedical applications. TGA analysis can be used to determine thermostability to investigate if the material can withstand elevated temperatures or not and the extent to which it can do so. On the other hand, XRD is essential to obtain an indication of crystallinity, since the size is important for the interaction between biomaterial and cells. Therefore, the adhesion can be directly affected. Furthermore, FTIR can identify the functional groups which also directly affect the adhesion between the material and specific cells. That behavior is due to the charged functional groups which can adhere to the oppositely charged cell components. The optical properties might support the correlation between the incident light and the bandages.

It has been reported that skin cancer is mainly treated by surgery. In this case, the potential bacterial invasion is one of the major challenges after surgery, which might delay healing and might cause negative circumstances. In this case, the acceleration of the wound healing could be a simple strategy to avoid the persistent growth of residual cancer after surgery. Hence, dual-functional natural-based films which could inhibit cancer cell growth and promote normal cell integration are highly preferred. In this case, CS doped with ZnS, ZrO_2_, and/or GO with significant biocompatibility could be suggested for wound/cancer treatment.

The major objective of this article is to find a new and easy technique for accelerating the healing process using a polymer filled with nanoparticles of ZnS or ZrO_2_, with/without GO nanoparticles. This work will also characterize the structural, morphological, thermal, and optical properties of the synthesized polymer-based films.

## 2. Materials and Methods

### 2.1. Materials

The zinc sulfide (30–80 nm) (99.98%), zirconium oxide (40–70 nm) (99%), acetic acid (99%), and chitosan (98.0%) with high molecular weight (300,000 g/mol) were bought from Sigma-Aldrich (St. Louis, MO, USA). Moreover, GO was prepared in the laboratory by following a modified Hummers’ method [1,2].

### 2.2. Preparation of Scaffold with Different Contents of Oxides

The CS films were formed using the casting methodology. In order to obtain the film solutions, 1 g of CS was dissolved in 100 mL of solvents containing 98 mL of deionized water and 2 mL of acetic acid. The precise concentration of the CS solution was 1.0 wt. %. The (100 mL) stock solution of CS was divided into 5 bottles, and the bottles were numbered from 1 to 5, each one containing 20 mL of CS solution. The total amounts of the powder ingredients (ZnS, ZrO_2_, and GO) were injected through the CS solution at a fixed ratio of 20 wt. % for each sample. In other words, the precise quantity in the solution for each sample/bottle contains 0.2 g of CS doped with 0.04 g of the powder ingredients. The first sample (bottle no. 1) was pure CS without additional powder ingredients. The second one (bottle no. 2) was made with an additional 0.04 g of ZnS, while the third (bottle no. 3) contained 0.04 g of ZrO_2_. The fourth sample (bottle no. 4) contained 0.04 g of both ZnS and ZrO_2_, while the last one (bottle no. 5) contained 0.035 g of both ZnS and ZrO_2_ as well as 0.05 g of the GO mixture. The prepared mixture solutions were stirred for 4 h at room temperature to obtain well-dispersed solutions. The solutions were poured onto 5 glass plates with a diameter of 14 cm. They were dried in the drier furnace for 24 h at 50 °C.

### 2.3. Characterizations

#### 2.3.1. XRD Measurements

X-ray diffraction (XRD) analyses were carried out via the X-ray diffractometer model (analytical-x’ pertpro carried out using Cu kα1 radiation, λ = 1.5404 Å, 45 kV, 40 mA, The Netherlands). It was mainly used to investigate the phase composition of the as-synthesized film composite. All XRD curves of the final materials were scanned in the range of 5° ≤ 2θ ≤ 70° with a step size of 0.02° and a step time of 0.5 s.

#### 2.3.2. FTIR Measurements

Fourier-transformed infrared (FTIR) spectra were scanned with an FTIR spectrometer (Perkin-Elmer 2000, Waltham, MA, USA) in the range of 4000–400 cm^−1^. The preparation stages were completed as follows: Obtaining around 2 g of the powder. The sample was mixed in a mortar with KBr at a ratio of 20:1. The sample was placed in a holder and compressed hard so that it adhered to the sample perfectly. Finally, the sample was placed in an FTIR instrument and scanned in the range of 400 to 4000 cm^−1^.

#### 2.3.3. Examination of Film Morphology and EDX

FESEM was used to investigate the morphology of WI-38 cells on the film-based NC. For this purpose, the nano-film was sanitized using a UV lamp for 30 min. Each sample was cropped into two pieces measuring 0.5 × 0.5 cm, then they were placed into a 12-well plate. A total of 1.5 mL of WI-38 cells was added to each well. The plate was then incubated at 37 °C for 3 days. After this time, the films were washed with phosphate-buffered saline (PBS). To keep the cells fixed on the film surface, the scaffolds were submerged in a glutaraldehyde (98%) solution (2.5% *v*/*v*) for 1.0 h. Then, they were dehydrated in the air for ¼ h. Finally, they were coated with gold for just 2 min to be ready for the resultant FESEM surface images. The EDX device is connected to the FESEM apparatus. Therefore, the EDX device was used to analyze the elemental structure after taking the SEM micrograph.

#### 2.3.4. Thermogravimetric Analysis

TGA was carried out from room temperature up to 600 °C in a thermal analyzer (DTG-60H SHIMADZU, Tokyo, Japan) using an airflow rate of 100 mL/min. The heating rate was 10 °C/min.

#### 2.3.5. UV Measurements

Double beam UV–visible (UV–Vis) (Shanghai Metash Instruments Co., Shanghai, China) spectroscopy was used to investigate the optical properties of the polymeric samples. All compositions were examined. Each sample was cropped to 2 cm × 2 cm. All samples were examined in film form. The thickness of these films was maintained at 0.25 mm. The thickness was measured using a digital caliper. The measurement of the adsorption behaviour was repeated three times for the polymeric films, while the standard deviation was calculated using Stata Statistical Software (StataCorp. 2019. StataCorp LLC, College Station, TX, USA).

### 2.4. Contact Angle

A small piece of each film was cropped to a size of 1 cm × 1 cm. It was fixed to the front of a small camera on a horizontal plate. A drop of deionized water was dropped on the horizontal film, while the camera was used manually to take one image for each drop on the film. The camera used was a “1600× 8 LED Digital USB Microscope Magnifier Electronic Stereo USB Endoscope Camera, Shenzhen Yuan Qi Trading Co., Ltd., Shenzhen, China”. The images were taken manually 1 s after the addition of the drop. The image obtained was then introduced one by one to the “HiView” software to measure the contact angle from the right and left sides of each droplet image. Then, the values of the right and left angles were gathered and divided by 2 to get their average value. This process was repeated for each sample three times. In other words, three images were taken for each sample to proceed with the statistical analysis. The standard deviation was obtained for each sample using the Stata Statistical Software (StataCorp. 2019. StataCorp LLC, College Station, TX, USA).

### 2.5. In Vitro Cell Viability Tests

The in vitro test was carried out in VACSERA Co. in Giza, Egypt. The WI-38 cell lines were isolated from the lung tissue of a 3-month-old female embryo (accession Numbers: WI-38 (ATCC CCL-75); organism: Homo sapiens, human; cell type: fibroblast; tissue: lung; age: 3 months’ gestation; gender: female; morphology: fibroblast; growth properties: adherent; disease: normal). The normal lung cells (WI-38) were used under culture conditions in Dulbecco’s modified Eagle’s medium (DMEM, Gibco) to investigate cell viability. Cells with a density of 5 × 103 (cells/cm^2^) were cultured on the films using 24-well plates; then, they were incubated at 37 °C. After 3 days of incubation, the media was detached and MTT (3-(4,5-dimethylthiazol-2-yl)-2,5-diphenyltetrazolium bromide) was injected into each well; then, cell viability was measured with an optical analyzer.

## 3. Results and Discussion

### 3.1. Structural Investigation

**Figure 1:**
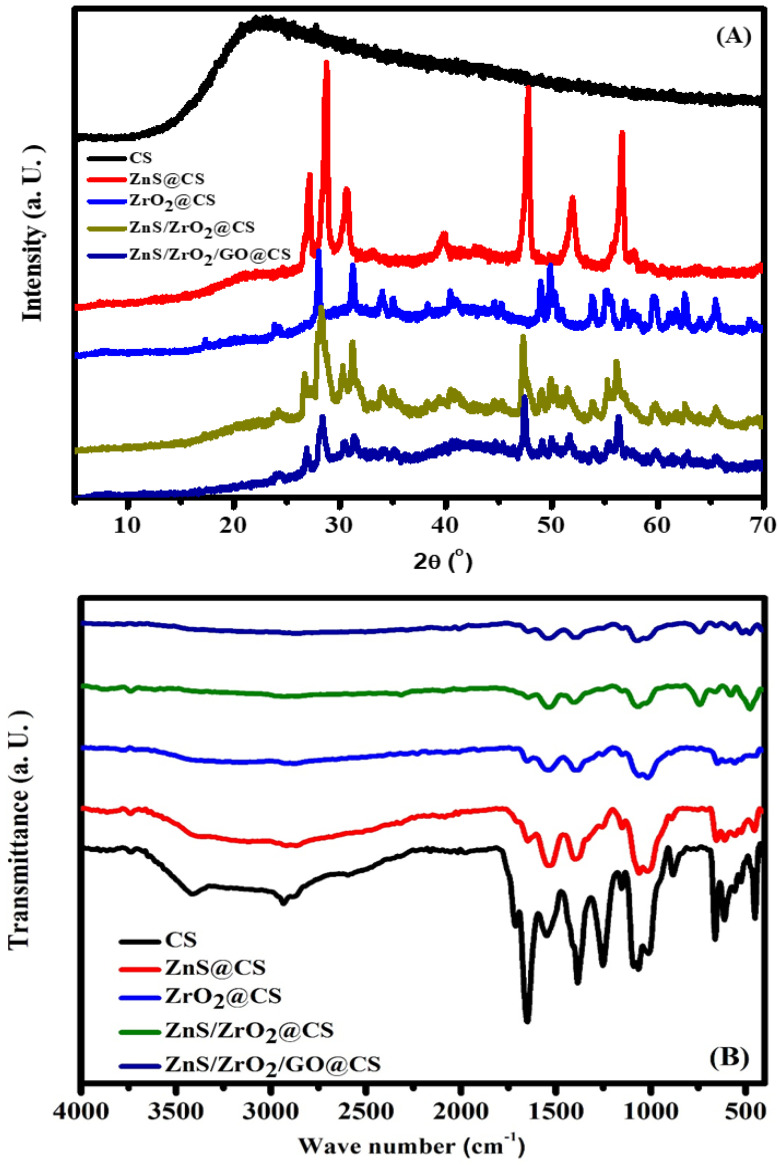
Structural investigation using XRD (**A**) and FTIR (**B**) spectra of CS-based NC films.

The XRD diffractogram that appears in Figure 1A shows the CS phase via the intense peaks at 2θ = 21.6° [3,4]. The indicated broad CS peak corresponds to a single phase of amorphous CS [3,4]. Prior studies have reported that H-bonds (intramolecular and intermolecular) are the cause of the rigid structure of CS [5]. Generally, XRD of GO nanostructures shows a characteristic peak at 2θ = 10.2 [6]. The diffractogram of the ZnS/ZrO_2_/GO@CS NC film demonstrates the disappearance of the main GO peak owing to the disturbance of its 2D structure with the remaining 3D constituents [7]. Additionally, the main diffraction peaks of ZnS are positioned at 2θ = 27.1, 47.9, and 56.35° [8]. On the other hand, the diffractograms of monoclinic ZrO_2_ (according to JCPDS: 01-088-2390) display peaks at 27.8°, 31.2°, and 34.1° [9]. Indeed, the sharp peaks provide evidence for the crystalline nature of the tested oxy components [10]. Figure 1A also showed the pure compositional components of ZnS/ZrO_2,_ GO, and CS, and demonstrated that the introduced XRD bands are an integration of the raw constituents’ main peaks. The XRD plots show no general features that correspond to crystalline/amorphous phases [11]. Furthermore, the chitosan FTIR spectra contributed several intense vibrational bands corresponding to CN–NH, CH_3_, and CH_2_–OH, which were displayed in the 1240–1370 cm^−1^ range [12]. The band detected at 1647.1 cm^−1^ can be assigned to the stretching vibration of the carbonyl group (C=O), whilst the band at 1073 cm^−1^ corresponds to the stretching vibrational motion of –C–O–C [13]. In addition, the amide group vibrational band is located around 1372 [12]. The bands at 2934 cm^−1^ represent the stretching vibration of C-H [12]. The bands at 3416 cm^−1^ might be equivalent to the vibrational mode of O-H due to the intramolecular H-bonding [14]. Regarding the carbon-based GO ingredient spectra, the frail peaks at 1720 cm^−1^ (ƲC-O) might signify that the addition of ZrO_2_ and ZnS ingredients causes a partial reduction of GO to reduced-GO (rGO) [8]. Similarly, the ZnS distinguishing peaks are shown in the 450∓660 cm^−1^ scope, which agrees with the fingerprint for the metal sulfide bond. Thus, peaks at 468, 578, and 657 cm^−1^ can be ascribed to ZnS components in the NC films [8]. In Figure 1B, the FTIR spectra also confirm the insertion of ZrO_2_. Overall, the broad peak at 3600–3100 cm^−1^ specified the presence of the OH group that is created from the precursor intermediate (Zr-OH) via its stretching vibration oscillations [14]. In addition, the previous data confirmed the presence of the pure compositional constituents (ZnS/ZrO_2,_ GO, and CS) whose FTIR peaks are integrated with the raw constituents’ characteristic peaks.

### 3.2. EDX

**Figure 2:**
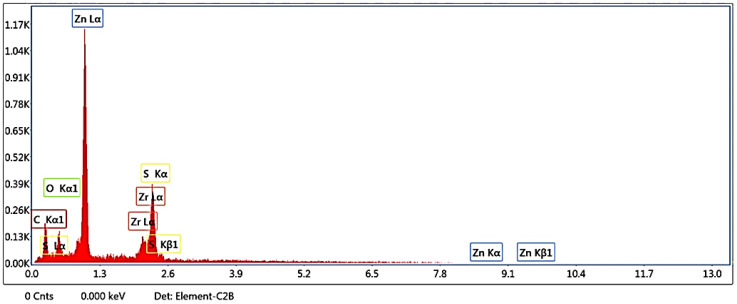
EDX of ZnS/ZrO_2_/GO@CS film.

EDX analysis contributed quantitative and qualitative data for the ZnS/ZrO_2_/GO@CS NC film (Figure 2). The quantitative inspection of inserted elements is performed by analyzing the signal intensities [15]. The data in Table 1 offer the properties of the C, O, Zn, Zr and S elements with atomic % of 59.44, 13.45, 17.07, 0.57, and 9.47%, respectively. The oxygen element appears at 0.5 keV, as demonstrated in Figure 2 [16]. The Lα, Kα, and Kβ characteristic peaks of Zn are positioned at 1.1, 8.6 and 9.5 KeV, respectively. Additionally, the Kα of the C-atom band appears at 0.3, while the band at 2.4 Kev can be attributed to the sulfur element. Likewise, the resultant data also confirm the high purity of the FSC-based NC (ZnS/ZrO_2_/GO@CS).

### 3.3. Morphological Study

**Figure 3:**
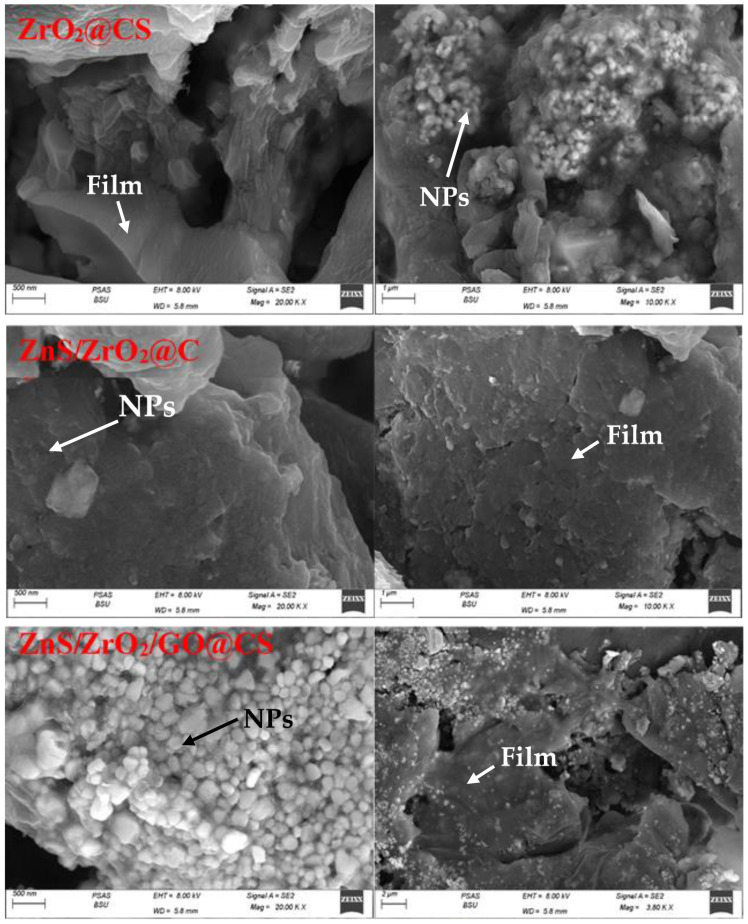
SEM micrographs of CS-based nanofilms.

The topographical and elemental imaging of the ZrO_2_@CS composition is displayed in Figure 3. The SEM micrograph shows zirconium oxide grain aggregates with a polymeric sheath around them. The macroporous structure reveals an average pore size of 570 nm. Porosity is a crucial factor in the healing process and it makes passing nutrients easier by permitting vascularization [17]. The average ZrO_2_ grain size is 87 nm. It is noticed that the particle size of ZrO_2_ is larger than that of the precursor ones, which is attributed to the agglomeration tendency of these particles when they pass through the polymeric films. Additionally, the insertion of ZnS to form the ZnS/ZrO_2_@CS composition causes a significant variation in the overall topographical features, although the macroporous features are maintained. The circular ZrO_2_ grains show a scattered appearance while maintaining their range of shapes and sizes. Moreover, the ZnS/ZrO_2_/GO@CS film shows a significant widening of the introduced surface area, along with the preservation of its porous structure and the improvement in the spreading of inorganic compositions between its sheets upon addition of the polymeric components. The average ZrO_2_ grain size is 96 nm. Thus, the introduced surface area and porosity can be considered a function of the chemical composition. Furthermore, it could be optimized by varying the quantity and type of constituents.

### 3.4. Wettability

**Figure 4:**
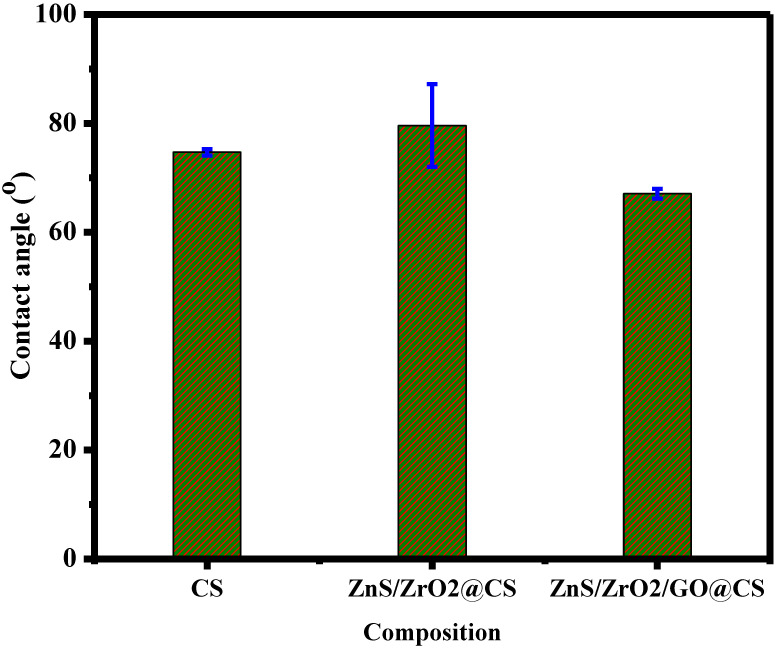
Wettability of CS-based nanofilms.

The measured contact angle for the CS composition is 74.7 ± 0.6°, while the binary ZnS/ZrO_2_@CS film reaches an angle of 79.6 ± 7.6°; finally, ZnS/ZrO_2_/GO@CS corresponds to the lowest angle of 67.1 ± 0.9°. The pattern that appears in Figure 4 indicates that ZnS/ZrO_2_/GO@CS has higher potential for biocompatibility. Moreover, the lower contact angle offers greater adhesion potential; in brief, the chemical composition directly impacts the surface polarity, contact angle value, and adhesion capability [18]. The contact angle increased from its value in pure CS to the highest value in ZnS/ZrO_2_@CS film and decreased after the addition of GO. The hydrophobic behavior has been lowered with the incorporation of GO, which is accompanied by the crosslinking of the oxygenated groups in GO layers with the surrounding molecules. The lowering of hydrophobicity occurs in parallel to the development of hydrophilic behavior, which is essential to support the chemical reactivity towards the neighboring environment. Surface adhesion is supposed to occur via two main mechanisms: physical linking and chemical bonding. Physical cohesion seems to be predominant in light of the notches and roughness associated with the polymeric film. The roughness is accompanied by the hydrophilic tendency of the scaffolds. Thus, the incorporation of nanoparticles into the films induces high roughness, promoting hydrophilicity, which might support the bioactivity of the film towards the host tissues. The chitosan contact angles reported by BUMGARDNER et al., 2014 l matches well with the contact angles of the chitosan-coated surfaces experimentally measured in the present work (76.4 ± 5.1°) [19].

### 3.5. Thermal Study

**Figure 5:**
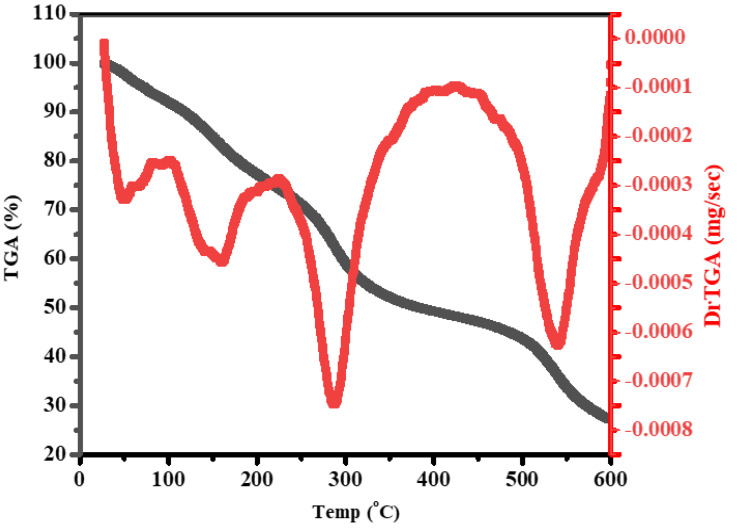
Thermal behavior of ZnS/ZrO_2_/GO@CS film.

The thermal behavior of ZnS/ZrO_2_/GO@CS film was studied by identifying the decrease in weight of the tested composition that occurred with a linear elevation in temperature using thermogravimetric analysis (TGA). The TGA setting conditions were a temperature rate of 10 °C/min with a temperature range of 30–600 °C under N_2_ gas. Figure 5 demonstrates the TGA/DTA plot with overlapping degradation steps and four temperature peaks. The range of the first decomposition step is 30–105 °C/Ts: 50 °C, followed by 105–225 °C/Ts: 160 °C, then 225–420 °C/Ts: 288 °C, while the 420–588 °C/Ts: 540 °C step leaves 27% as a residue owing to the relatively low amount of mineral oxides inserted into the tested composition. The high stability of the incorporated metal oxide (ZrO_2_) and sulfate (ZnS) might support this hypothesis. It could be reported that the pure ZrO_2_ might transform into its allotropic structure at temperatures over 1100 °C, which provide the CS-containing films the ability to resist high temperature. Pure CS seems to be have lower thermal stability than the modified films, especially with these highly stable nanoparticles. Nevertheless, the weight loss beneath 25 °C corresponds to the structural-bound water [20]. The stage that occurs from 225 to 420 °C represents the thermal degradation of an organic portion (chitosan) [21]. The GO fragmentation stages involve two steps occurring from 100 °C to around 350 °C, which can be attributed to oxy-group fragmentations [22]. Additionally, Mattevi et al. reported the conversion of GO to rGO at 450 °C or above, which is equivalent to a chemical reduction process carried out by hydrazine monohydrate at 80 °C followed by heating at 200 °C to produce GO honeycomb [23].

### 3.6. Optical Behavior

The main parameter that summarizes the optical behavior of substances is the band gap. The band gap is detected by examining the absorption spectrum. However, a direct measurement of optical absorption is challenging due to the structures of tested compositions, i.e., nontransparent powders, compact disks, and ceramics [24]. The bands at 230 and 252 nm confirm the π-π* and n-π* transitions of C=C and C=O. Moreover, the vertical straight plot in the visible region denotes the transparency of the studied polymer [25]. Originally, the peak in this region is referred to as the n→ π* transition. The appearance of several bands as a result of the rest of the ingredient insertions at 358, 422, 555, and 585 nm confirm the occurrence of the mixing tactic and the slight deviations could be explained by the low contributions of ZnS, ZrO_2_, and GO.

**Figure 6:**
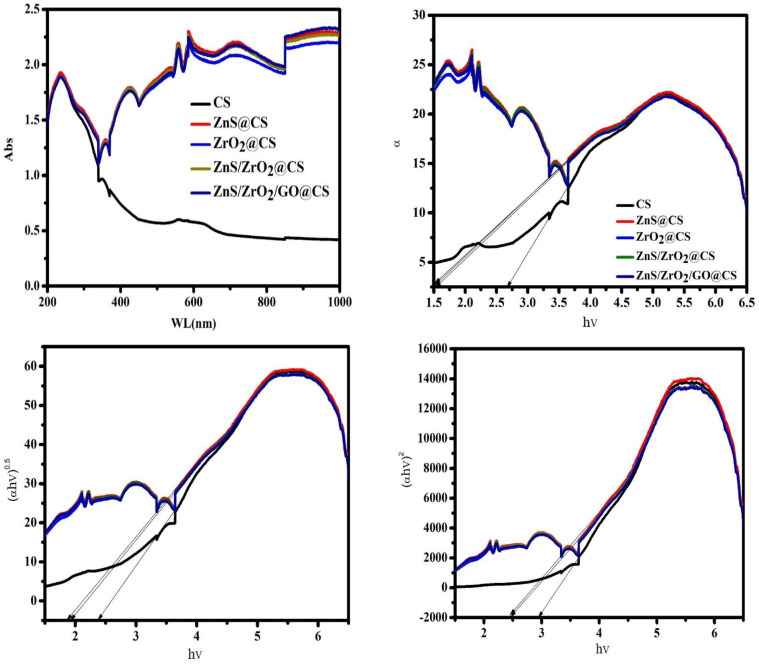
Optical behavior of CS-based polymeric films.

It is interesting to consider the applicability of polymers with inorganic additive films by studying their optical features. The optical absorption coefficients (*α*) are obtained by Beer–Lambert’s equation [26]:

(1) $$\alpha \left(\lambda \right)=\frac{2.303\;A}{d}\;$$

where (*A*) is for absorbance and (*d*) is for the film’s thickness. As noticed, α is plotted as a function of photon energy (*hν*). By changing the inorganic constituent inserted (ZnS, ZrO_2_, and GO), the absorption edge moves along the x-axis. An amount of 2.35 eV is recorded for CS, while it declines to be within the range of 1.8:1.9 by mixing the fabricated inorganic/organic films, as shown in Table 2. Furthermore, the band gap is calculated using equation [27,28]:

(2) $$\alpha h\nu =A{\left(h\nu -Eg\right)}^{m}$$

where *Eg* is the band gap and A refers to the band tailing parameter. The power (*m*) is a direct transition if *m* = 0.5 and an indirect transition if *m* = 2. The optical band gaps are found by taking the intercept values of the extrapolated linear part of the plots, as shown in Figure 6 [29]. In addition, structural alterations were confirmed by changing the *E_g_* values, as mentioned in Table 2. Additionally, the energy gaps of pure ZrO_2_ and ZnS are about 5.5 and 3.7 eV, respectively [30,31]. Thus, the data revealed in Table 2 show a great reduction in the gap energy of the pure raw ingredients by virtue of the mixing tactic. The Dimitrov and Sakka equation is used to find the refractive index (*n*) as follows:

(3) $$\frac{n^{2}-1}{n^{2}+1}=1-\sqrt{\frac{Eg_{i}}{20}}$$

where $Eg_{i}$ is an indirect energy band gap. The refractive index of CS is 3.577 ± 0.23, while it was recorded as 2.186 ± 0.21 for ZnS@CS, ZrO_2_@CS, and ZnS/ZrO_2_@CS. Additionally, insertion of GO into the ternary film causes a minor increase in the band gap, reaching 2.171 ± 0.26. Thus, the fabricated films, which vary in terms of thickness and in the raw materials used, exhibit optical deviations. The decreasing band gap of the fabricated films from 2.35 ± 0.19 to 1.9 ± 0.15 eV for pure CS and ZnS/ZrO_2_/GO@CS, as mentioned in Table 2, indicates that significant changes in the optical properties occurred upon the addition of nanoparticles. The interaction between the incident light and the fabricated films is highly important to induce a form of charge carriers through the layers of films. These generated charges involving electrons and holes might play an important role in the activation of direct chemical interactions between the embedded nanoparticles of ZnS/ZrO_2_ and/or GO and the host tissues. Thus, the low band gaps could represent a simple strategy to promote drug delivery through the wound site. The injury solution could act as a medium to deliver ions from the bandages to the host tissues. Hence, the optical properties could work hand in hand with the surface features that enhance the reactivity of the wound dressing via physical interlocking and ionic trapping enabled by the surface defects.

### 3.7. Selectivity Performance of ZnS/ZrO_2_/GO@CS in Reacting with Normal and Malignant Cell Lines

**Figure 7:**
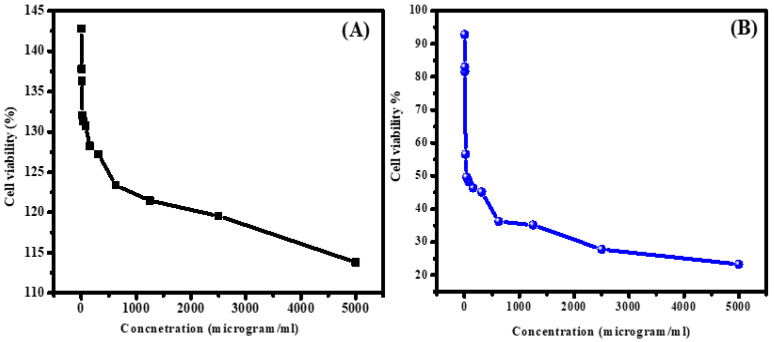
Cell viability of ZnS/ZrO_2_/GO@CS film (**A**) using normal lung cells; (**B**) anticancer activity against fibroblast cancerous cells.

**Figure 8:**
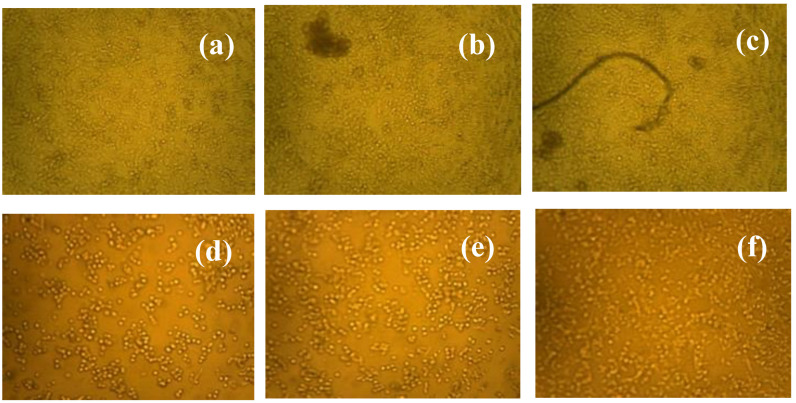
Optical microscope images clarifying the different concentrations of ZnS/ZrO_2_/GO@CS. (**a**–**c**) Cell viability of the normal lung cells (AI-38) after the incubation period, while the cells were exposed to a concentration of (**a**) 4000 µg/mL, (**b**) 2000 µg/mL, or (**c**) 500 µg/mL. (**d**–**f**) Cell viability of the cancer lung cells (A549) after the incubation period, while the cells were exposed to a concentration of (**d**) 4000 µg/mL, (**e**) 2000 µg/mL, or (**f**) 500 µg/mL. (The magnification is maintained at 100×).

The selective impact of ZnS/ZrO_2_/GO@CS on normal and malignant cells is revealed in this section. Figure 7 displays a plot showing the effect of ZnS/ZrO_2_/GO@CS concentration on normal and malignant cell lines. In comparing the resultant viability of malignant cells with that of normal cells, inspection of the growth of normal lung cells (WI-38) demonstrated that the use of 2.4 µg/mL leads to a cell viability % of 142.79%, while 5000 µg/mL leads to a viability of 113.82%. The resultant viabilities exceed the expected results, which points to the negligible toxicity of ZnS/ZrO_2_/GO@CS, as shown in Figure 8. On the other hand, the malignant cells that were exposed to 2.4 µg/mL showed a viability % of 92.81%, and this percentage shows a steep decline to 82.97% when the concentration of ZnS/ZrO_2_/GO@CS is raised to 4.9 µg/mL. Likewise, the usage of 5000 and 2500 µg/mL cause a great decline in the cell viability % of abnormal cells, reaching 23.28 and 27.81%, respectively. Bai et al., 2019 established a pH-responsive scaffold (GO-PCL) in which GO is improved by functionalizing it with gambogic acid (GA). In the acidic media that surrounds a cancerous bulk, GA could be discharged from the GO-PCL composition and attached to the Hsp90 protein, causing a mild temperature change for cancer cells without affecting normal cells; additionally, the mortality of MCF-7 breast cancer cells reaches >95% in vitro [32]. Thus, the selective behavior is tightly related to the environmental pH of the tissues. A. Nithya et al. prepared CS-Cu NCs and examined their antimicrobial and anti-cancer cell activity. They reported that the inhibition of A549 cancerous cells reached 10% after 24 h of incubation. Furthermore, the zone of inhibition against S. pneumonia bacteria reached around 20 mm [33]. Furthermore, J. Venkatesan et al. prepared nanocomposites containing porous CS-alginate biosynthesized silver nanoparticles. They found anticancer activity for the NCs and the population of apoptotic cells reached around 20% [34]. Moreover, N. Arjunan et al. prepared biogenic CS-Ag NCs and they reported their antimicrobial and anticancer activity. With respect to the antimicrobial activity, the zone of inhibition against Staphylococcus aureus reached around 18 mm. In addition, the anticancer activity reached around 90% inhibition (i.e., the viability of cancerous cells was 10%) at a concentration of around 100 µg/mL [35]. I. Salcedo et al. prepared nanocomposites based on CS. They reported a cell viability of around 70% after 24 h of incubation with human colorectal adenocarcinoma cell lines [36]. Moreover, S. Hsu et al. prepared CS-Au NCs and they found that wound enclosure reached around 100% after 14 days and around 75% after 7 days [37]. From the previous studies, it can be seen that pure CS seems to be biocompatible with biological environments owing to its natural source and safe fragments. In addition, the additional nanoparticles have not only maintained the biocompatibility of the CS, but they also contribute to the increase in biocompatibility, as obvious in Figure 7. On the other hand, the anticancer functionality of the fabricated films is initiated via the encapsulated nanoparticles, which tend to have high reactivity with cellular proteins. The degradation components of the embedded nanoparticles (ZnS, ZrO_2_, and GO), including reactive oxygen species (ROS), might be activated against cancer cells.

## 4. Conclusions

In this work, ZnS/ZrO_2_/GO components are merged with a chitosan film structure with the aim of achieving promising biological performance in the wound healing process. The morphological inspection by SEM demonstrates the formation of porous films with a good scattering of ZnS and ZrO_2_ nano-grains, especially among the ZnS/ZrO_2_/GO@CS film. In addition, ZnS/ZrO_2_/GO@CS exhibits the lowest contact angle of 67.1 ± 0.9°. Regarding the optical behavior, the absorption edge reaches 2.35 eV for chitosan, while it declines to a value within the range of 1.8–1.9 when it is merged with ZnS, ZrO_2_, and GO. Inspection of the growth and proliferation of a normal lung cell line demonstrates that the use of 2.4 µg/mL ZnS/ZrO_2_/GO@CS leads to a cell viability of 142.79%, while the use of 5000 µg/mL leads to a viability of 113.82%. In contrast, the fibroblast cancerous cell line exposed to 2.4 µg/mL ZnS/ZrO_2_/GO@CS reaches a viability of 92.81%, and this percentage shows a steep decline with the use of 5000 µg/mL, reaching 23.28%. Auxiliary biological evaluation of the fabricated film scaffolds reveals medically promising selective activity.

## Figures and Tables

**Table 1:** Results of the EDX analysis of ZnS/ZrO_2_/GO@CS film.

Element	Error %	Weight %	Atomic %
C K	13.66	29.74	59.44
O K	14.99	8.96	13.45
ZnL	3.59	46.47	17.07
ZrL	12.13	2.18	0.57
S K	5.56	12.65	9.47

**Table 2:** Optical properties of chitosan-based films, including the absorption edge, direct/indirect band gaps, and refractive index (*n*).

Composition	Thickness (mm) ± SD	Absorption Edge (eV) ± SD	Band-Gap (eV)		*n* ± SD
			Direct ± SD	Indirect ± SD	
CS	0.66 ± 0.12	2.35 ± 0.11	2.35 ± 0.19	2.9 ± 0.23	3.577 ± 0.23
ZnS@CS	0.12 ± 0.07	1.5 ± 0.17	1.8 ± 0.10	2.4 ± 0.19	2.186 ± 0.21
ZrO_2_@CS	0.13 ± 0.09	1.55 ± 0.19	1.85 ± 0.21	2.4 ± 0.17	2.186 ± 0.19
ZnS/ZrO_2_@CS	0.06 ± 0.01	1.55 ± 0.13	1.85 ± 0.13	2.4 ± 0.22	2.186 ± 0.17
ZnS/ZrO_2_/GO@CS	0.17 ± 0.04	1.6 ± 0.09	1.9 ± 0.15	2.45 ± 0.09	2.171 ± 0.26

## Data Availability

The raw/processed data generated in this work are available upon request from the corresponding author.
